# Re-emergence of the susceptibility of the *Salmonella* spp. isolated from blood samples to conventional first line antibiotics

**DOI:** 10.1186/s13756-016-0121-8

**Published:** 2016-05-25

**Authors:** Krishma Laxmi Shrestha, Narayan Dutt Pant, Raju Bhandari, Sabita Khatri, Basudha Shrestha, Binod Lekhak

**Affiliations:** Department of Microbiology, Goldengate International College, Battisputali, Kathmandu, Nepal; Department of Microbiology, Grande International Hospital, Dhapasi, Kathmandu, Nepal; Department of Microbiology, Kathmandu Model Hospital, Kathmandu, Nepal

**Keywords:** Enteric fever, *Salmonella* spp., First line antibiotics, Fluoroquinolones, Antibiotic resistance, Nepal

## Abstract

**Background:**

Enteric fever is an important public health problem in Nepal. Due to emergence of multidrug resistant strains of *Salmonella* spp. the conventional first-line drugs, ampicillin, chloramphenicol, and cotrimoxazole have not been used as empiric therapy for treatment of enteric fever for last two decades and there have been increased uses of fluoroquinolones as the drugs of choice. The aim of this study was to evaluate and analyze the antimicrobial susceptibility patterns of *Salmonella* spp.

**Methods:**

A total of 620 blood samples collected from the patients suspected of suffering from enteric fever were cultured using standard microbiological techniques. Antibiotic susceptibility testing of the *Salmonella* spp., was performed by Kirby Bauer disc diffusion technique following Clinical and Laboratory Standard Institute (CLSI) guidelines. Minimum inhibitory concentrations of ciprofloxacin, ofloxacin and nalidixic acid were determined by agar dilution method.

**Results:**

Of the total 83 *Salmonella* spp., 48 (57.83 %) were *S. Typhi* and 35 (42.26 %) were *S. Paratyphi A*. Among 83 *Salmonella* isolates, 98.8 % of the *Salmonella* spp. were susceptible to chloramphenicol and co-trimoxazole and about 97.6 % of the isolates were susceptible to ampicillin. Similarly, 69 (83.13 %) isolates were resistant to nalidixic acid. Only 16.9 % of the isolates were susceptible to ciprofloxacin. One *S. Typhi* isolate was multidrug resistant.

**Conclusion:**

The present study revealed the decreased susceptibility of the *S. Typhi* and *S. Paratyphi A* to fluoroquinolones, proving them to be inappropriate for empirical therapy for the treatment of enteric fever in our setting. Further the higher susceptibility of the isolates to first line drugs, ampicillin, chloramphenicol, and cotrimoxazole suggests the possibility of using these drugs for empirical therapy.

## Background

Enteric fever is an infection caused by *Salmonella enterica* serotype *Typhi* and *Paratyphi* [1]. It has been estimated that there are worldwide, approximately 22 millions typhoid cases and 200,000 deaths per year [2]. *Salmonella enterica* serovar *Paratyphi A* causes additional 5·4 millions illnesses [2]. Enteric fever, is a serious public health problem in Nepal and is attributed to fecal contamination of foods and drinking water [3].

Since 1989, multidrug resistant strains of *Salmonella*, those are no longer susceptible to the first line antibiotics have emerged [4]. Multidrug resistant *Salmonella enterica* strains (resistant to chloramphenicol, ampicillin, cotrimoxazole) are common in Asia [5]. During the past two decades, more than 80 % of the known multidrug resistant strains of *Salmonella* have been reported from Pakistan, Iran, Nepal, Bangladesh and India [6, 7]. And the fluoroquinolones have been suggested as the drugs of choice for the treatment of the enteric fever caused by species of *Salmonella* resistant to first line antibiotics [8].

Over the last decade there have been several enteric fever epidemics in Nepal [9, 10] with changing antibiotic resistance patterns [11]. The multidrug resistant *S. Typhi* in Nepal was first reported in 1991 [12]. Subsequently, with the introduction of fluoroquinolones for the treatment of enteric fever, nalidixic acid-resistant strains associated with reduced susceptibility to fluoroquinolones have been increasingly reported from Nepal [10]. And the use of third generation cephalosporins and azithromycin as treatment options for enteric fever has increased [13]. There have been reports of occasional isolation of the highly fluoroquinolone resistant and third generation cephalosporin resistant strains of *S. Typhi* and *S. Paratyphi A* from Nepal [14, 15]. And recently the re-emergence of the conventional first-line drugs susceptible strains of *Salmonella* spp. in Nepal has been reported (Susceptibility of *Salmonella Typhi* to chloramphenicol and cotrimoxazole was 100 % and that to ampicillin was 98.2 %. Similarly, 100 % of *S. Paratyphi A* strains were sensitive to cotrimoxazole and ampicillin with 96.7 % of the isolates being sensitive to chloramphenicol) [13]. In the present situation, when the treatment options for enteric fever are decreasing, the re-emergence of strains of the *Salmonella* susceptible to ampicillin, chloramphenicol, and cotrimoxazole should be evaluated to find out their therapeutic importance [13]. For timely proper management of the enteric fever the knowledge of the prevalence of the different serovars of *Salmonella* and their antimicrobial susceptibility patterns is of utmost importance [13].

In this study we studied the antimicrobial susceptibility patterns of *Salmonella Typhi* and *Salmonella Paratyphi A* isolated from the blood samples of the patients suspected of suffering from enteric fever toward different commonly used antibiotics. Reduced susceptibility of nalidixic acid-resistant strains to fluoroquinolones was also evaluated.

## Methods

A cross-sectional study was carried out from November 2012 to May 2013, at a tertiary care hospital in Kathmandu, Nepal. A total of 620 patients clinically suspected of suffering from enteric fever, were included in the study. Patients who had already received antibiotics were not included in the study. Blood samples were collected aseptically by vein puncture and inoculated immediately into brain heart infusion broth. The amount of blood collected from adults was 5 ml and that from children was 2 ml. The ratio of amount of blood to that of brain heart infusion broth was maintained to be 1:10. After incubation, at 37 °C for 24, 48 and 72 hrs subcultures were done on MacConkey agar and blood agar and were observed for bacterial growth after 24 hrs of aerobic incubation at 37 °C. Isolates were identified by biotyping (colony morphology, staining reaction and biochemical characteristics) and serotyping using specific antisera (Denka Seiken Co. Ltd, Tokyo, Japan) [16]. Samples were considered negative for *Salmonella*, if no growth was observed on subculture after 7 days of aerobic incubation at 37 °C. Antimicrobial susceptibility testing for *Salmonella* serovars was performed by Kirby Bauer disc diffusion technique following CLSI guidelines [17]. The antibiotic discs used were, ampicillin (30 μg), nalidixic acid (30 μg), ofloxacin (5 μg), ciprofloxacin (5 μg), chloramphenicol (30 μg), ceftriaxone (30 μg), cotrimoxazole (1.25 μg), cefotaxime (30 μg), azithromycin (15 μg), and cefixime (30 μg). Minimum inhibitory concentrations of nalidixic acid, ofloxacin and ciprofloxacin were determined by agar dilution method as suggested by Andrews [18] following CLSI guidelines [17]. *Escherichia coli* ATCC 25922 was used for quality control. Statistical analysis was performed by using SPSS 19.0.

## Results

Out of 620 blood samples, 83 (13.38 %) samples were culture positive for *Salmonella* spp. Out of total 83 *Salmonella* spp. isolated, 48 (57.83 %) were *Salmonella Typhi* and 35 (42.26 %) were *Salmonella Paratyphi A*.

### Antimicrobial susceptibility patterns of *Salmonella* spp. toward different commonly used antibiotics

Among the antibiotics used for susceptibility testing of the isolates, all the isolates were found to be susceptible to cefixime, ceftriaxone, cefotaxime and azithromycin. Similarly, 98.8 % of the *Salmonella* spp. were susceptible to chloramphenicol and co-trimoxazole and about 97.6 % of the isolates were susceptible to ampicillin and ofloxacin. Only 16.9 % of the isolates were found to be susceptible to ciprofloxacin and nalidixic acid. 79.5 % of the isolates were intermediate sensitive to ciprofloxacin. Only one isolate was found to be multidrug resistant (showing resistance to ampicillin, chloramphenicol, cotrimoxazole and nalidixic acid) (Table 1).

**Table 1:** Antimicrobial susceptibility patterns of *Salmonella* spp. toward different commonly used antibiotics

Antibiotics	Number	Resistance (%)	Intermediate (%)	Sensitive (%)
Ampicillin	83	2.4	0	97.6
Ceftriaxone	83	0	0	100
Cefotaxime	83	0	0	100
Cefixime	83	0	0	100
Nalidixic acid	83	83.1	0	16.9
Ciprofloxacin	83	3.6	79.5	16.9
Ofloxacin	83	0	2.4	97.6
Cotrimoxazole	83	1.2	0	98.8
Azithromycin	83	0	0	100
Chloramphenicol	83	1.2	0	98.8

### Minimum inhibitory concentrations of nalidixic acid, ciprofloxacin and ofloxacin for *Salmonella* isolates

About 24.1 % of the isolates had minimum inhibitory concentration of nalidixic acid (≥ 16 μg/ml) and that of ciprofloxacin (≥ 0.5 μg/ml) in resistant range. Similarly, 59 % of the isolates had minimum inhibitory concentration of nalidixic acid (≥ 16 μg/ml) in resistant range but that of ciprofloxacin (0.064 μg/ml to 0.5 μg/ml) in intermediate susceptible range. Again, 16.9 % of the isolates had minimum inhibitory concentration of nalidixic acid (≤ 16 μg/ml) and that of ciprofloxacin (≤ 0.064 μg/ml) in susceptible range (Table 2). Likewise, 1.2 % of the isolates had minimum inhibitory concentration of ofloxacin (≥ 1 μg/ml) and that of nalidixic acid (≥ 16 μg/ml) in resistant range. Approximately, 69.9 % of the isolates had minimum inhibitory concentration of ofloxacin (≤ 0.125 μg/ml) in susceptible range while that of nalidixic acid (≥ 16 μg/ml) in resistant range. Around, 12 % of the isolates had minimum inhibitory concentration of ofloxacin (0.125 μg/ml to1 μg/ml) in intermediate susceptible range but that of nalidixic acid (≥ 16 μg/ml) in resistant range. Finally, 16.9 % of the isolates had minimum inhibitory concentration of nalidixic acid (≤ 16 μg/ml) and that of ofloxacin (≤ 0.125 μg/ml) in susceptible range (Table 3).

**Table 2:** Susceptibility of the *Salmonella* spp. toward nalidixic acid and ciprofloxacin on the basis of minimum inhibitory concentration

Nalidixic acid and ciprofloxacin resistant (%)	Nalidixic acid resistant and ciprofloxacin intermediate (%)	Nalidixic acid and ciprofloxacin sensitive (%)
24.1 %	59 %	16.9 %

**Table 3:** Susceptibility of the *Salmonella* spp. toward nalidixic acid and ofloxacin on the basis of minimum inhibitory concentration

Nalidixic acid and ofloxacin resistant (%)	Nalidixic acid resistant and ofloxacin sensitive (%)	Nalidixic acid resistant and ofloxacin intermediate (%)	Nalidixic acid and ofloxacin sensitive (%)
1.2 %	69.9 %	12 %	16.9 %

## Discussion

Similar rate of culture positivity as in our study (13.38 %) was also found in another study conducted in Nepal (15.6 %) [19]. Typhoid and paratyphoid, collectively known as ‘enteric fever’, remains as one of the commonest causes of the fever in most parts of under developed world including Nepal [20]. The enteric fever is endemic in most of the urban areas of Nepal including Kathmandu, mainly due to cross contamination of the drinking water with sewage [10, 21]. As in our study higher prevalence of *S*. Typhi (57.83 %) in comparison to *S*. Paratyphi A (42.26 %) (*p* < 0.05) was also reported in another study conducted in Nepal {*S*. Typhi (64.1 %) and *S*. Paratyphi A (35.9 %)} [22]. In both studies *S*. Paratyphi B and C were not isolated.

In present study, the rate of nalidixic acid resistance, which is a phenotypic marker for reduced susceptibility to fluoroquinolones [23]; was observed high (83.1 %). Similar rates of nalidixic acid resistance were also reported in other studies conducted in India (86.27 %) [24] and Nepal (91.1 % for *S*. Typhi and 90.0 % for *S*. Paratyphi A) [13]. In contrast to nalidixic acid resistance, in present study re-emergence of susceptibility (98.8 % susceptibility to chloramphenicol and co-trimoxazole and 97.6 % susceptibility to ampicillin) to conventional first line drugs used for treatment of enteric fever was observed. Similar types of findings were also observed by Garg et al. (1.96, 15.6, and 6.8 % resistance to chloramphenicol, ampicillin, and co-trimoxazole respectively) [24], Acharya et al. (95.12 % susceptibility to all first line antibiotics) [25], and Acharya et al. (more than 95.5 % susceptibility to all first line antibiotics) [26]. The increased use of the fluoroquinolones and the discontinuation in the use of the conventional first line antibiotics (ampicillin, chloramphenicol, and co-trimoxazole) for treatment of the enteric fever for long periods of time may be the reason behind the reduced susceptibility of *Salmonella* strains to fluoroquinolones and re-emergence of first line antibiotics susceptible *S. Typhi* and *S. Paratyphi A* isolates. Further, the loss of the plasmids responsible for resistance to first line drugs may be the reason for the re-emergence of the susceptible strains [13].

Among fluoroquinolones higher percentage of susceptibility was shown toward ofloxacin (97.6 %) followed by ciprofloxacin (16.9 %). But we do not recommend to use ofloxacin for the treatment of enteric fever in our setting, as high rate of nalidixic acid-resistance among *Salmonella* isolates was noted in our study, which indicates the high probability of developing ofloxacin resistance in near future, if the drug is used haphazardly. In Nepal, all the *S. Typhi* and *S. Paratyphi A* isolates were reported as susceptible until 1998 but during 1999 to 2003 ciprofloxacin resistance increased to 5 % in the *S*. Typhi and 13 % in S. Paratyphi A [9].

In contrast to our study, 100 % susceptibility of *Salmonella Typhi* and 96.7 % susceptibility of *Salmonella Paratyphi A* to ciprofloxacin were observed in a study by Chand et al. [13]. The increased haphazard use of ciprofloxacin as empirical therapy for treatment of enteric fever in recent years may have contributed to this discrepancy. Among cephalosporins and macrolides all the isolates were susceptible to cefotaxime, cefixime, ceftriaxone and azithromycin. But among the cephalosporins, cefixime may attract more attention as drug of choice as it can be used orally [27]. Only one isolate was found to be multidrug resistant in present study and the finding correlated with the previous study that reported the decreasing trend in multidrug resistant isolates during the period of 2000–2004 [28]. Due to irrational use of antibiotics, the rate of drug resistance among bacteria is increasing and the situation is worse in developing countries [29, 30]. So antibiotics should be used only on the basis of culture and sensitivity report.

Our findings will be helpful for the clinician to choose the appropriate empirical therapy for the treatment of enteric fever. Further it will also be helpful to make policy for empirical therapy for treatment of enteric fever.

### Limitations of the study

Due to short duration of the time available for research and lack of resources we could not include more samples in the study. Further, due to lack of sophisticated laboratory and lack of fund we could not confirm our results with molecular techniques. Minimum inhibitory concentrations of all the antibiotics used in antimicrobial susceptibility testing could not be determined. This is a study conducted in only one hospital, so multi-centered study covering wide geographical area would have generated more significant result.

## Conclusions

The present study revealed the increased rate of nalidixic acid resistant *Salmonella* spp. associated with reduced susceptibility to fluoroquinolones in contrast to increased susceptibility of the strains to conventional first-line drugs ampicillin, chloramphenicol, and cotrimoxazole in Kathmandu, Nepal. So, the conventional first-line drugs along with the third generation cephalosporins and azithromycin can be used as empiric therapy for treatment of enteric fever in our setting. Multi-centered studies covering wide geographical area and large population are required to generate more significant data regarding the susceptibility of the *Salmonella* spp. toward ampicillin, co-trimoxazole and chloramphenicol and to determine the possibility of using these drugs for empirical therapy for treatment of enteric fever.

## Abbreviations

ATCC, American Type Culture Collection; CLSI, Clinical and laboratory Standards Institute; SPSS, Statistical Package for the Social Sciences.

## References

[1] Parry CM, Thuy CT, Dongol S, Karkey A, Vinh H, Chinh NT (2010). Suitable disk antimicrobial susceptibility breakpoints defining Salmonella enterica serovar Typhi isolates with reduced susceptibility to fluoroquinolones. Antimicrob Agents Chemother.

[2] Crump JA, Luby SP, Mintz ED (2004). The global burden of typhoid fever. Bull World Health Organ.

[3] Pokharel P, Rai SK, Karki G, Katuwal A, Vitrakoti R, Shrestha SK (2009). Study of enteric fever and antibiogram of Salmonella isolates at a Teaching Hospital in Kathmandu Valley. Nepal Med Coll J.

[4] Threlfall EJ, Ward LR, Rowe B (1992). Widespread occurrence of multiple drug-resistant Salmonella Typhi in India. Eur J Clin Microbiol Infect Dis.

[5] Parry CM. Epidemiological and clinical aspects of human typhoid fever: in Salmonella infections clinical, immunological and molecular aspects. In: Mastroeni P, Maskell D, editors. Advances in molecular and cellular microbiology 9. New York: Cambridge University Press; 2006. pp. 1–17.

[6] Mohanty S, Renuka K, Sood S, Das BK, Kapil A (2006). Antibiogram pattern and seasonality of Salmonella serotypes in a North Indian tertiary care hospital. Epidemiol Infect.

[7] Menezes GA, Harish BN, Khan MA, Goessens WHF, Hays JP (2012). Antimicrobial resistance trends in blood culture positive Salmonella typhii solates from Pondicherry, India, 2005–2009. Clin Microbiol Infect.

[8] World Health Organization (2003). Background document: the diagnosis, treatment and prevention of typhoid fever. WHO/V&B/03.07.

[9] Maskey AP, Basnyat B, Thwaites GE, Campbell JI, Farrar JJ, Zimmerman MD (2008). Emerging trends in enteric fever in Nepal: 9124 cases confirmed by blood culture 1993–2003. Trans R Soc Trop Med Hyg.

[10] Lewis MD, Serichantalergs O, Pitarangsi C, Chuanak N, Mason CJ, Regmi LR (2005). Typhoid fever: a massive, single point source, multidrug resistant outbreak in Nepal. Clin Infect Dis.

[11] Malla S, Dumre SP. Changing trend of antimicrobial resistance toward Salmonella isolates of Nepal: findings of antimicrobial resistance surveillance program, Nepal. 13th International congress on Infectious Diseases (ICID), Kuala Lumpur, Malaysia, 19–22 June. 2008.

[12] Watson JP, Pettibone EC (1991). Chloramphenicol and ampicillin resistant Salmonella Typhi in Nepal. J Nepal Med Assoc.

[13] Chand HJ, Rijal KR, Neupane B, Sharma VK, Jha B (2014). Re-emergence of susceptibility to conventional first line drugs in Salmonella isolates from enteric fever patients in Nepal. J Infect Dev Ctries.

[14] Shirakawa T, Acharya B, Kinoshita S, Kumagai S, Gotoh A, Kawabata M (2006). Decreased susceptibility to fluoroquinolones and gyrA gene mutation in the Salmonella enterica serovar Typhi and Paratyphi A isolated in Katmandu, Nepal, in 2003. Diagn Microbiol Infect Dis.

[15] Chau TT, Campbell JI, Galindo CM, van Nguyan MH, Diep TS, Nga TTT (2007). Antimicrobial drug resistance of Salmonella enterica serovar Typhi in Asia and molecular mechanism of reduced susceptibility to fluoroquinolones. J Clin Microbiol.

[16] Cheesbrough M (2006). District laboratory practice in tropical countries, part II.

[17] Clinical Laboratory Standards Institute (CLSI) (2012). CLSI document M100S-S22. Performance standards for antimicrobial susceptibility testing: twenty second informational supplement ed.

[18] Andrews MJ (2001). Determination of minimum inhibitory concentration. J Antimicrob Chemother.

[19] Easow JM, Joseph NM, Dhungel BA, Chapagain B, Shivananda PG (2010). Blood stream infections among febrile patients attending a Teaching Hospital in Western Region of Nepal. AMJ.

[20] Maskey AP, Day JN, Tuan PQ, Thwaites GE, Campbell JI, Zimmerman M (2006). Salmonella enteric serovar Paratyphi A and S. enteric serovar Typhi cause indistinguishable clinical syndromes in Kathmandu, Nepal. Clin Infect Dis.

[21] Prajapati B, Rai GK, Rai SK, Upreti HC, Thapa M, Singh G (2008). Prevalence of Salmonella Typhi and Paratyphi infection in children: a hospital based study. Nepal Med Coll J.

[22] Adhikari D, Acharya D, Shrestha P, Amatya R (2012). Ciprofloxacin susceptibility of Salmonella enteric serovar Typhi and Paratyphi A from blood samples of suspected enteric fever patients. Int J Infect Microbiol.

[23] Crump JA, Barrett TJ, Nelson JT, Angulo FJ (2003). Re-evaluating fluoroquinolone breakpoints for Salmonella enterica serotype Typhi and for non-typhi salmonellae. Clin Infect Dis.

[24] Garg N, Tiwari R, Gupta V, Kapil A, Kumar S, Rishi P (2013). Current antibiogram and clonal relatedness among drug-resistant Salmonella enteric serovar Typhi in Northern India. Microb Drug Resist.

[25] Acharya D, Malla S, Adhikari N, Dumre SP (2012). Current fluoroquinolone susceptibility criteria for Salmonella needs re-evaluation. Kathmandu Univ Med J.

[26] Acharya D, Trakulsomboon S, Madhup SK, Korbsrisate S (2012). Antibiotic susceptibility pattern and the indicator of decreased ciprofloxacin susceptibility of Salmonella enteric serovar Typhi isolated from Dhulikhel Hospital Nepal. Jpn J Infect Dis.

[27] Thaver D, Zaidi AK, Critchley JA, Azmatullah A, Madni SA, Bhutta ZA. Fluoroquinolones for treating typhoid and paratyphoid fever (enteric fever). Cochrane Database Syst Rev. 2008;4:CD004530. 10.1002/14651858.CD004530.pub3 18843659

[28] Khanal B, Sharma SK, Bhattacharya SK, Bhattarai NR, Deb M, Kanungo R (2007). Antimicrobial susceptibility patterns of Salmonella enterica serotype Typhi in Eastern Nepal. J Health Popul Nutr.

[29] Neupane S, Pant ND, Khatiwada S, Chaudhary R, Banjara MR (2016). Correlation between biofilm formation and resistance toward different commonly used antibiotics along with extended spectrum beta lactamase production in uropathogenic Escherichia coli isolated from the patients suspected of urinary tract infections visiting Shree Birendra Hospital, Chhauni, Kathmandu, Nepal. Antimicrob Resist Infect Control.

[30] Awasthi TR, Pant ND, Dahal PR (2015). Prevalence of MultiV drug resistant bacteria in causing community acquired urinary tract infection among the patients attending outpatient Department of Seti Zonal Hospital, Dhangadi, Nepal. Nepal J Biotechnol.

